# Nerve‐on‐a‐Chip Derived Biomimicking Microfibers for Peripheral Nerve Regeneration

**DOI:** 10.1002/advs.202207536

**Published:** 2023-04-29

**Authors:** Yunru Yu, Binghui Jin, Jinghao Chen, Chenghao Lou, Jiahui Guo, Chaoyu Yang, Yuanjin Zhao

**Affiliations:** ^1^ Department of Rheumatology and Immunology Nanjing Drum Tower Hospital School of Biological Science and Medical Engineering Southeast University Nanjing 210096 P. R. China; ^2^ Wenzhou Institute University of Chinese Academy of Sciences Wenzhou Zhejiang 325000 P. R. China; ^3^ Oujiang Laboratory School of Pharmaceutical Sciences Wenzhou Medical University Wenzhou Zhejiang 325035 P. R. China

**Keywords:** bioinspired, hydrogel, microfiber, microfluidics, nerve regeneration, organ‐on‐a‐chip

## Abstract

Fibrous scaffolds have shown their advantages in tissue engineering, such as peripheral nerve regeneration, while most of the existing fiber‐shaped scaffolds are with simple structures, and the in vitro performance for nerve regeneration lacks systematic analysis. Here, novel nerve‐on‐a‐chip derived biomimicking microfibers for peripheral nerve regeneration are presented. The microfibers with controllable core–shell structures and functionalities are generated through capillary microfluidic devices. By integrating these microfibers into a multitrack‐architectured chip, and coculturing them with nerve cells as well as gradient bioactive elements, the nerve‐on‐a‐chip with the capabilities of systematically assessing the performances of nerve fiber formation in the hollow microfibers at in vitro level is constructed. Based on a rat sciatic nerve injury model, the rapid promotion ability is demonstrated of optimized microfibers in nerve regeneration and function recovery in vivo, which implies the credibility of the nerve‐on‐a‐chip on biomimicking microfibers evaluation for peripheral nerve regeneration. Thus, it is convinced that the organ‐on‐a‐chip will undoubtedly open up a new chapter in evaluating biological scaffolds for in vivo tissue engineering.

## Graphical abstract

Microfibers with desired structures are generated and integrated into a chip with multitrack, cocultured with nerve cells and gradient extracellular matrixes to form the nerve‐on‐a‐chip. The nerve‐on‐a‐chip is able to systematically compare and assess the performances of nerve fiber formation in the hollow microfibers at in vitro level. A rat sciatic nerve injury model is used to demonstrate its credible assessing and screening ability.

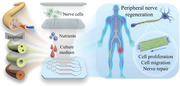

## 1 Introduction

Peripheral nerve injury has become such a common but severe problem in the daily life of patients that it calls for efficient interventions or therapies.^[^ ^1^ ^]^ Existing approaches like autografts, allografts, and chemical or electrical stimulations have fortunately gained gratifying achievements, while these remedies usually face limitations or risks, including chances of neuroma formation, second trauma, lack of donor sites, and many others.^[^ ^2^ ^]^ As alternatives, emerging tissue engineering materials such as fibrous scaffolds or microtubes have attracted numerous research attention in damaged nerve repair, regeneration, or replacement because they can provide topographical guidance for axonal outgrowth during nerve regeneration.^[^ ^3^ ^]^ Although with several successful examples, the implantable nerve tissue‐engineered materials still lack a systematic and biomimetic preclinical evaluation platform to observe their repair performance at in vitro level, which would lead to limited healing efficiency and even unforeseen inflammation. In addition, these materials are often with homogeneous morphologies or simple functions that are not versatile enough to meet complex implantation requirements. Thus, implantable materials with desired cavity structures for nerve regeneration as well as the preclinical assessing platform are worth anticipation.

**Figure 1:**
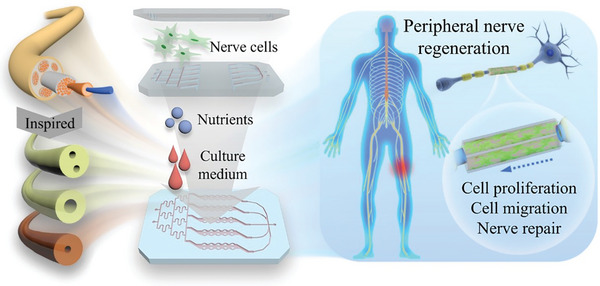
Schematic illustration of nerve‐on‐a‐chip derived biomimicking microfibers and their applications in peripheral nerve generation. Inspired by the microstructure of the nerve, several hollow microfibers were generated via microfluidic spinning and integrated with a multichannel chip. In this chip, microfibers were immobilized in the bottom layer, the cell culture medium with gradient nutrients could be injected, and the nerve cell suspension was introduced to the hollow channel of the fiber through the middle layer channels. The fibers for nerve regeneration could thus be analyzed by observing the cell culture condition in the chip.

Herein, we present a novel nerve‐on‐a‐chip derived biomimicking microfiber for peripheral nerve regeneration, as schemed in **Figure** **1**. Among various tissue engineering materials, fibrous scaffolds have shown their great attributes in nerve regeneration due to their nerve fiber‐mimicking structure.^[^ ^4^ ^]^ To fabricate these microfibers, especially hollow microfibers, the microfluidic spinning approach has shown its competitiveness due to the convenient adjustment in channel architectures and flow parameters.^[^ ^5^ ^]^ However, the microstructures of these microfibers are far from the native fibrous tissues, which could not fully meet the demanding requirements in the 3D cell culture and in vitro nerve models rebuild. In addition, the nerve regeneration performances of these microfibers before their in vivo applications lack systematic comparison and evaluation. In contrast, organ‐on‐a‐chip systems have achieved great progress in online biological analysis.^[^ ^6^ ^]^ These elaborate platforms construct in vitro architecture, and simulate metabolic activities in extracellular microenvironments, providing many brand‐new models for healthy or diseased organs, studying physiological mechanics, and screening toxicity of drugs at the in vitro level.^[^ ^7^ ^]^ Although they have succeeded in bridging the gap between predicting drug performance in humans and in vivo animal experiments, these systems are seldom used in assessing and screening implantable tissue‐engineered materials, especially those for peripheral nerve regeneration.

In this paper, taking advantage of microfluidic spinning in microfiber generation and organ‐on‐a‐chip in native tissue context mimicking, we present nerve‐on‐a‐chip derived microfibers for peripheral nerve regeneration (Figure 1). During this process, hollow microfibers with controllable structures and functionalities were fabricated by capillary microfluidic devices based on their administrable flow choice and designable microchannel configurations. The microfibers were integrated into a multitrack architecture chip and cocultured with nerve cells as well as gradient extracellular matrixes (ECM) to form the nerve‐on‐a‐chip, which made it possible to systematically compare and assess the performances of nerve fiber formation in the hollow microfibers at in vitro level. Thus, microfibers with appropriate and effective capabilities for nerve regeneration could be screened out via this nerve‐on‐a‐chip. It was demonstrated through a rat sciatic nerve injury model that the optimized microfibers could facilitate the rebuilding and function recovery of nerve fiber in vivo, which demonstrated the credible assessing and screening ability of the nerve‐on‐a‐chip. These results indicated that the proposed nerve‐on‐a‐chip is valuable in cultivating and evaluating biological scaffolds for in vivo regenerations, which will undoubtedly open up a new chapter of the organ‐on‐a‐chip in tissue engineering fields.

## 2 Results and Discussion

**Figure 2:**
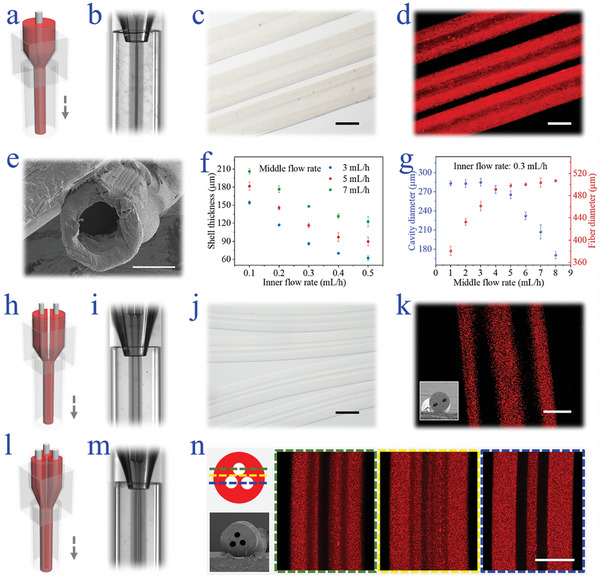
Microfluidic preparation of multiple types of microfibers. a) The scheme illustrates the spinning of hollow microfiber. b) Real‐time photograph of the hollow microfiber generation. c) Microscopic image of hollow microfiber in the bright field. d) Fluorescent microscopic image of the hollow microfiber. e) SEM image showing the fiber cross‐section. f) Relationship between shell thickness and inner flow rate under different middle flow rates. g) Relationships between outer flow rates and cavity as well as fiber diameter. h) Scheme of the microfluidic spinning of the dual‐channel fiber (DCF). i) Online observation of DCF spinning. j) Microscopic image of DCF. k) Image of the vertical section of the DCF characterized by a confocal laser scanning microscopy (CLSM), and the inserted figure is an SEM image showing the cross‐sectional region of the DCF. l) Scheme of the configuration of the microfluidic device used for triple‐channel fiber (TCF) spinning. m) Online image of the spinning process of TCF. n) General view and SEM image of TCF and corresponding vertical views of the fiber. Scale bars are (c) 500 µm, (d) 500 µm, (e)100 µm, (j) 500 µm, (k) 200 µm, and (n) 200 µm, respectively.

In a typical experiment, the microfluidic chip for hollow microfiber preparation was made by constructing a spindle, a tapered, along with a cylindrical capillary sequentially into a square one, named the inner, middle, outer, and observing channel, respectively (**Figure** **2**a). To fabricate microfibers with hollow structures, the sheath fluid of calcium chloride (CaCl_2_), the middle flow of the sodium alginate and Gelatin‐Methacryloyl (Alg‐GelMA) mixture, and core CaCl_2_ fluid were infused into the outer, tapered, and spindle microchannels successively. During the microfluidic spinning process, a coaxial flow was shaped due to the low Reynolds number and the hydrodynamic effect in the microfluidic device (Figure 2b). The hollow structure of the microfiber was then formed benefitting from the laminar flow pattern and fast diffusion‐controlled ion cross‐linking process of alginate together with the photo‐crosslinking of GelMA at both the inner and outer surface of the middle flow. With continuous diffusion and cross‐linking, the hollow hydrogel microfiber could be extruded and collected from such a microfluidic spinning process.

After cleansing with deionized water twice, the collected microfibers were observed under the optical microscope. To facilitate the observation of their structure, red fluorescent microparticles were dispersed in the Alg‐GelMA mixture. The resulting microfibers were observed in cylindrical shapes, and sharp interfaces of both inner and outer surfaces could be seen (Figure 2c,d). Specifically, the shell of the microfiber was uniform, which was also demonstrated by the scanning electron microscopic (SEM) image (Figure 2e). The versatility of microfluidic spinning imparted the fiber with controllable and adjustable structures. For these hollow microfibers, the tunable shell thickness could be achieved under different inner and middle flow rates. It demonstrated that the increase in inner flow rates resulted in a decrease in shell thickness, while that in middle flow rates would induce the shell thickness increase (Figure 2f). This effect can be ascribed to the diameter change in the hollow core and the whole fiber. By increasing the middle flow rate while keeping the inner flow rate constant, the cavity diameter decreased sharply and the microfiber diameter increased obviously, as shown in Figure 2g.

Capillary microfluidics offers the advantage of easy customization of microfiber structures by simply modifying the injection microchannels. To illustrate this, two spindle injection capillaries were inserted into the middle injection channel in parallel to endow the microfiber with two hollow channels, termed dual‐channel fiber (DCF) (Figure 2h). Thanks to the laminar flow behavior, two inner fluids could flow parallelly inside the middle flow, which precisely duplicated the configuration of microchannels (Figure 2i). It is similar to the formation of hollow microfiber in that fast ion‐induced cross‐linking of alginate took place at the interfaces of inner and middle flows to generate the DCF in situ. The channels inside were observed equal and uniform because of the stable flows during the microfluidic spinning (Figure 2j,k). Additionally, microfiber with three channels inside, named triple‐channel microfiber (TCF) could also be achieved by introducing the third spindle injection channel to the microfluidic device used for DCF preparation (Figure 2l). Similarly, the fluids replicated the configuration of three paralleled injection microchannels when they met at the taper of the middle channel and flowed inside the collection channel (Figure 2m). The SEM image of the microfiber demonstrated three homogeneous and center symmetrically aligned cavities. This structure was also confirmed by the cross‐sectional laser scanning microscopic characterizations (Figure 2n).

Apart from the tailorable morphologies, the microfiber could also be equipped with active characteristics. Because conductive microfibers have shown their potential in nerve injury treatments, the possibility of microfluidic spinning in the preparation of conductive microfibers was investigated. In this regard, graphene oxide (GO) nanosheets were introduced into the middle Alg‐GelMA mixture. It could be seen that different addition of GO nanosheets would not interfere with the synthesis of microfiber with one or two cavities via microfluidic spinning (Figure S1, Supporting Information). The microfibers showed hollow structures and the brown color indicated the successful encapsulation of GO. Similarly, different flow rates and GO contents could also impart the microfiber with varied hollow channel diameters and electrical conductivities (Figure S2 and S3, Supporting Information). It could be concluded that the microfiber with condensed shell thickness and extended length showed an increased resistance, which reflected the decrease in conductivity (Figure S4, Supporting Information).

The biological safety of the resultant microfibers and their ability for nerve cell migration guiding were studied. After 24 and 72 h of coculturing Schwann cells, a principle glia cell of the peripheral nerve system, with the obtained hollow microfibers, the CCK‐8 array was conducted to indicate that both the DCF and double‐channel GO microfiber (DCGF) showed great biocompatibility (Figure S5, Supporting Information). It is also worth mentioning that both of the microfibers promoted cell proliferation compared to the control group. Besides, the ability to guide nerve cell migration of DCF and DCGF was evaluated (Figure S6, Supporting Information). It demonstrated that both the DCF and DCGF groups had more migrated Schwann cells per field than the control group. Comparatively, the cell amount in DCGF was more than that in the microfiber without GO encapsulation group, indicating that the addition of GO would further promote the migration of Schwann cells. Before they were analyzed in the chip, the degradability of the alginate‐GelMA microfibers was studied (Figure S7, Supporting Information). It could be seen that after 8 weeks of treatment, both two types of hydrogels maintain half of their weight even under enzymolysis. Notably, the double‐network hydrogel, showed more weight remaining compared to the calcium alginate hydrogel, which revealed the benefit of additive GelMA.

**Figure 3:**
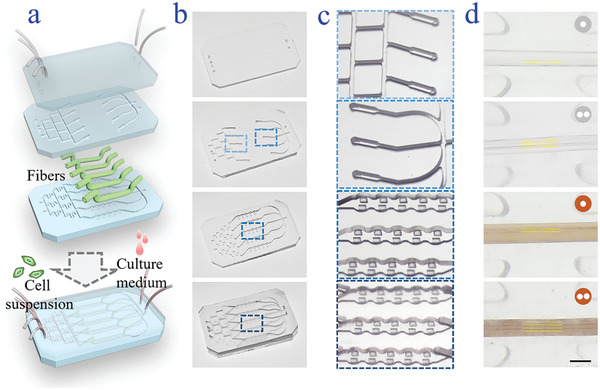
The integration between microfibers and the multilayered microfluidic chip. a) Schematic illustration of the construction of the multilayered microfluidic chip. b) Digital images of the upmost, middle, and bottom layers, and the assembled chip. c) Bright‐field microscopic image of the details in the microchannels, indicating the inlet and outlet of the middle layer, the microfiber stabilizing channel of the bottom layer, and the assembled microfiber stabilization as well as cell cultivation region, respectively. d) Optical microscopic images of the hollow microfiber, DCF, single‐channel GO microfiber (SCGF), and DCGF in the channel, respectively. The scale bar is 300 µm.

To systematically assess and compare the performances of the generated microfibers in nerve fiber formation, we designed a three‐layered nerve‐on‐a‐chip (**Figure** **3**a). By replicating the designed mold of each layer using highly transparent, biocompatible, and operative polydimethylsiloxane (PDMS), a three‐layered microfluidic chip could be constructed (Figure 3b,c). The upmost thin layer provided a transparent window for further observation and was taken as the cover of the nerve‐on‐a‐chip; The middle layer consisted of symmetrical injection and ejection channels for the introduction of cell suspensions into the hollow channel of the microfibers; The bottom layer was with multitrack and several columns of pillars configuration, in which microfibers could be inserted and fixed between the pillars. It is thus possible to realize the observation and comparison of performances of multiple microfibers in a controlled manner. The injection channels were utilized to introduce cultivation solutions, and the outlet was designed to eject the solutions containing metabolic productions and wastes for further analysis if necessary. After three layers were treated with plasma, the ends of microfibers were fixed in the channels of the middle layer, leaving the middle part beneath. The nerve‐on‐a‐chip could then be obtained by bonding each layer and keeping the middle part of the fiber inside the channel of the bottom layer. Although they showed some shrinkage, the microfibers maintained their core–shell structures and could be stabilized between the two columns (Figure 3d).

**Figure 4:**
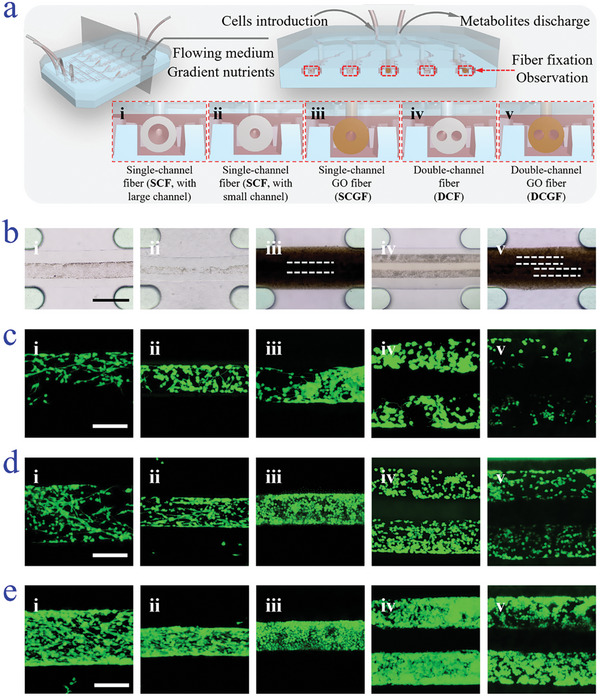
Cell proliferation of microfibers with different structures. a) Schematic illustration of the construction of nerve‐on‐a‐chip and cross‐section of fibers fixed inside the chip. b) Optical microscopic images of cells cultivated in (i) microfibers with large hollow channels, (ii) microfibers with small hollow channels, (iii) SCGF, (iv) DCF, and (v) DCGF. The scale bar is 800 µm. The hollow channels are around 400 µm in SCF with a large channel, and around 200 µm in SCF with a small channel, SCGF, DCF, and DCGF, respectively. c–e) Representative fluorescent images of formed cell bundles in five types of microfibers on (c) Day 1, (d) Day 3, and (e) Day 5, respectively. The scale bars are 200 µm.

During the cell cultivation, the cell suspensions were introduced into the hollow microfiber through injection channels of the middle layer. After the cells were seeded on the inner wall of the microfibers, the inlets could also be used for cell culture media injection. Additionally, the cell culture media with gradient nutrients were obtained via mixing two different concentrated cell culture media through the bifurcated small injection channels and flowed surrounding the microfibers in the channel to mimic in vivo microenvironment of cell growth (**Figure** **4**a). Since Schwann cell proliferation together with migration is a critical indicator in peripheral nerve regeneration, the nerve‐on‐a‐chip was used to study the proliferation of Schwann cells in different structured hollow microfibers. It was first used to study the cell proliferation behavior under different concentrations of ECM, which is a mixture of extracellular macromolecules and minerals, including collagen, fibronectin, laminin, etc., and is beneficial for cell adhesion, interaction, and growth. Benefitting from the bifurcated inlets of the bottom layer, a gradient concentration of ECM could be introduced to the chip by pumping two flows of ECM solutions with different concentrations into two inlets and mixing in furcated injection channels. It showed that the speed of cell proliferation in microfibers followed a concentration‐dependent manner (Figures S8 and S9, Supporting Information). Specifically, the increased concentration of ECM would facilitate cell proliferation and bundle formation in the hollow microfibers. After 5 d of cultivation, a continuous cell fiber could be observed inside the microfiber in a culture medium containing the highest concentration of ECM.

The nerve‐on‐a‐chip is a versatile tool that can be used to analyze various factors that affect nerve cell proliferation due to its highly integrated structure. In addition to studying the effect of ECM concentration, the chip can also be used to investigate the impact of GO on Schwann cell proliferation. It could be realized by integrating hollow microfibers encapsulating different concentrations of GO in each channel. As expected, the Schwann cells significantly proliferated and formed a bundle in the hollow microfiber with higher GO encapsulation (Figures S10 and S11, Supporting Information). Although the increase was not significantly different, it could still be observed that after 5 d of cultivation, the microfiber with the highest GO concentration contained more cells and the cell bundle is much denser. This may be attributed to the increased electrical conductivity of GO microfiber, which would facilitate the communication between nerve cells and thus promote their proliferation and migration to form the bundle.

The nerve‐on‐a‐chip platform demonstrated its potential as a 3D in vitro system for microfiber screening in nerve injury treatment by enabling the observation and analysis of cell proliferation in different cultivation environments. To this end, hollow microfibers with different hollow channel diameters, DCF, single‐channel GO microfiber (SCGF), and DCGF were embedded into each channel of the chip and cocultured with Schwann cells for 5 d. It was found that for all types of microfibers, the Schwann cells could proliferate and migrate in their channels with the addition of ECM in the culture media (Figure 4; Figure S12, Supporting Information). In detail, for microfibers with only one channel, those with thicker shells showed relatively enhanced promotion of cell proliferation due to space confinement (Figure 4b(i)–e(ii)). In addition, the increase in the channel amount would allow sufficient space for cells to be proliferated although the seeded cells in two channels were almost the same as that with one channel (Figure 4b(iv)–e(iv)). As a result, the DCF could form two bundles of nerve cells during 5 d of cultivation, which may be beneficial in nerve regeneration. Because it is not easy to observe the cell culture condition in the triple‐channel microfiber, and the increase of channel amount would not obviously affect the nerve formation speed, we only chose double‐channel microfiber here. It is worth mentioning that the addition of GO would promote such performance, where the encapsulated cells accelerated their migration to form aggregates during the cultivation (Figure 4b(iii)–e(iii),b(v)–e(v)). On this occasion, DCF and DCGF were expected to have a better treatment potential in peripheral nerve regeneration.

**Figure 5:**
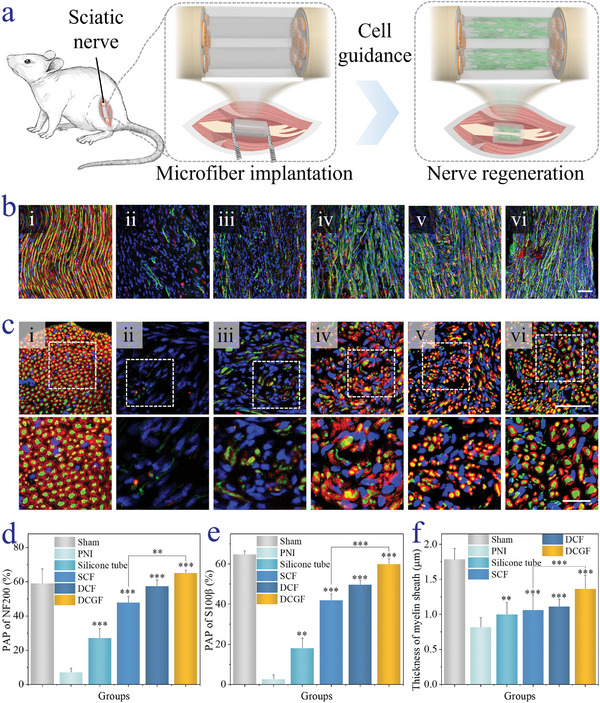
Axonal regeneration and remyelination studies. a) Schematic illustration of sciatic nerve injury model in SD rat and the treatment of microfiber. b) Representative photographs of NF‐200, S100*β*, and DAPI co‐staining of nerve longitudinal sections in (i) sham, (ii) PNI, (iii) Silicone tube, (iv) SCF, (v) DCF, and (vi) DCGF 8 weeks after the surgery. c) Representative images of NF‐200, MBP, and DAPI costained cross‐sectional sections of regenerated nerves in (i) sham, (ii) PNI, (iii) Silicone tube, (iv) SCF, (v) DCF, and (vi) DCGF 8 weeks postsurgery. The enlarged images are also shown. d,e) Statistical analysis of positive area percentage (PAP, %) of NF‐200, and S100*β* reacted for each group. f) Myelin sheath thickness analysis according to (c) of each group. The scale bars are (b) 50 µm, (c) 50 and 20 µm, respectively. (*n* ≥ 3, *p < 0.05, **p < 0.01, ***p < 0.001, versus PNI).

To prove the in vitro assessment of the nerve‐on‐a‐chip and demonstrate the clinical value of nerve‐on‐a‐chip derived microfibers in peripheral nerve regeneration, the microfiber candidates were transplanted into the 10 mm sciatic nerve defect models in SD rats (**Figure** **5**a). According to the nerve‐on‐a‐chip study, ECM was introduced to the middle flow during the microfiber spinning. After that, all of the generated hydrogel microfibers were then assembled in the medical silicone tube to prevent mechanical damage during the transplantation and maintain their straight morphology. The rats were randomly divided into six groups, namely, the sham, the peripheral nerve injury (PNI), the silicone tube, single‐channel microfiber with large channel (SCF), DCF, and the DCGF group. Since the sciatic function index (SFI) is a direct and reliable indicator to measure nerve function recovery, walking track analysis was implemented, and SFI was calculated at the 2nd, 4th, and 8th weeks (Figure S13, Supporting Information). Generally, PNI narrows both toe spread and intermediate toe spread, and results in low SFI, indicating a decrease in the function of sciatic nerves. After the treatment, it showed that the SFI improved during the regeneration, especially in the DCGF group. After 8 weeks post‐surgery, the sciatic nerve was exposed and its function recovery was investigated through an electrophysiology study. In detail, the compound action potential responding to the sciatic nerve stimulation induced at the proximal nerve stump above the transplanted site was measured and recorded (Figure S14a, Supporting Information). The compound action potential could be observed in groups of sham, PNI, silicone tube, SCF, DCF, and DCGF, illustrating the recovery of electrical function and formation of myelination (Figure S14b, Supporting Information). Especially, the conduction velocity and elicited amplitude significantly increased in the DCGF group, while the latency showed a decrease compared to other groups, which confirmed that the electrical conductivity of the medium is essential in nerve regeneration (Figure S14c–e, Supporting Information).

Additionally, axonal regeneration and remyelination were studied via immunofluorescence staining on both longitudinal and transversal sides. The regenerated nerve slices were stained by NF‐200, S‐100*β*, MBP, and DAPI, where NF‐200 indicated neurofilaments and axons, S‐100*β* specifically marked Schwann cells, MBP represented the myelination of the neuro fiber, and DAPI showed the cell nucleus. As shown in Figure 5b and Figure S15 (Supporting Information), NF‐200‐positive, and S‐100*β*‐positive cells were distributed in longitudinal sections of experimental groups, confirming the regeneration of the myelinated nerve fibers. Comparatively, the expression of NF‐200 and S‐100*β* positive protein upregulated in microfibers treated groups, indicating a better formation of axons and myelin in these groups (Figure 5d,e). It is worth noting here the regenerated nerve fibers in the DCGF group behaved much straighter compared to those in other groups. This should be attributed to the electrical conductivity and axon connection effect of conductive GO. The transversal immunofluorescence staining images also reflected that the nerve‐on‐a‐chip derived microfibers performed obvious nerve regeneration abilities (Figure 5c,f; Figure S16, Supporting Information). Taken together, the silicone tube would not show obvious side effects, and the nerve‐on‐a‐chip derived biomimicking microfibers showed favorable nerve regeneration stimulation behaviors.

**Figure 6:**
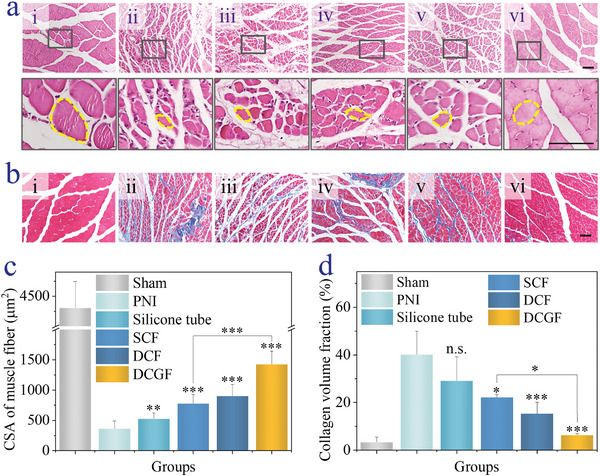
Muscle recovery investigation after the rats were treated with silicone tubes and microfibers. a) Representative H&E staining images of the cross‐sectional gastrocnemius muscle of (i) sham, (ii) PNI, (iii) Silicone tube, (iv) SCF, (v) DCF, and (vi) DCGF groups. b) Representative images of the cross‐sectional gastrocnemius muscle of (i) sham, (ii) PNI, (iii) Silicone tube, (iv) SCF, (v) DCF, and (vi) DCGF groups highlighted by Masson's trichrome stain. The blue staining indicates collagen fiber deposition and muscle fibrosis. c) Statistics showing the cross‐section of gastrocnemius muscles in each group. d) Collagen volume fraction percentage of gastrocnemius muscles in each group. The scale bars are 100 µm. (*n* ≥ 3, *p < 0.05, **p < 0.01, ***p < 0.001, n.s.: not significant, versus PNI).

The treatment results in different groups were intensively studied by gastrocnemius amyotrophy analysis, which was also an important manner to evaluate nerve recovery. Gastrocnemius atrophy was recorded through weight ratio (Figure S17, Supporting Information). It could be seen that the weight loss decreased in SCF, DCF, and DCGF groups, demonstrating an obvious nerve function recovery. This could be verified by intensively investigating cross‐sectional areas (CSAs) of muscle fibers and collagen fibers. After Hematoxylin‐Eosin (H&E) staining, the CSA of muscle fibers was observed and analyzed. As shown in **Figure** **6**a, apparent muscle atrophy could be seen in the PNI group, indicating a severe nerve injury. Among the treating groups, the CSA of muscle fibers was significantly elevated in the DCGF group, indicating the best nerve regeneration performance (Figure 6c). In addition, the collagen deposition around the atrophied muscle fibers was investigated by Masson's trichrome staining (Figure 6b). It statistically demonstrated that the average percentage of collagen fiber areas and muscle fibrosis detected in microfiber‐treated groups was less than that in PNI and silicone tube‐treated groups (Figure 6d). Specifically, the decrease was significantly shown in DCF and DCGF groups compared to the SCF group, indicating a better functional recovery. All of these suggested that the nerve‐on‐a‐chip‐derived microfiber treatment would improve muscle recovery.

## 3 Conclusion

In conclusion, we have presented nerve‐on‐a‐chip derived biomimicking microfibers for peripheral nerve regeneration. A series of microfibers with tailorable structures and functions were consecutively obtained from flexibly assembled capillary microfluidic chips, benefitting from the adjustable phase flow rates and composition of microfiber precursors. The microfibers, nerve cells, and culture media with bioactive additions were introduced into the multilayered and multitrack architecture to form the nerve‐on‐a‐chip. By providing a 3D microenvironment for nerve cells and creating a gradient concentration of bioactive additions in the cultivation medium, the nerve‐on‐a‐chip can systematically analyze the influence of ECM on the nerve cell fiber formation, and compare the performances of each integrated microfiber at in vitro level. Based on these, microfibers with appropriate and effective capabilities for nerve regeneration could be screened out. The credibility of the nerve‐on‐a‐chip was proved by applying the microfiber candidates to sciatic nerve‐injured rats, where the chosen microfibers rapidly promoted nerve fiber regeneration and functional recovery. Taking these features together, it is believed that the organ‐on‐a‐chip would certainly start a new avenue for the assessment of biological scaffolds for in vivo tissue engineering applications. It is also convinced that, by increasing the number of channels, a high throughput chip could be built for preliminary analysis of tissue‐engineered fibrous scaffolds. In addition, by introducing more complex configurations of solutions, different pressures, flow stresses, the influence of the surrounding environments on the cells, the interaction between cells and materials, and many others could be investigated based on such a versatile platform.

## Conflict of Interest

The authors declare no conflict of interest.

## Supporting information

Supporting Information

## Data Availability

The data that support the findings of this study are available from the corresponding author upon reasonable request.
