# Supplementary material for: Nerve‐on‐a‐Chip Derived Biomimicking Microfibers for Peripheral Nerve Regeneration
Source: Adv Sci (Weinh). 2023 Apr 29;10(20):2207536. doi: 10.1002/advs.202207536 (PMC10369236; doi:10.1002/advs.202207536)
Supplement: Supplementary file 1 — Supporting Information [file ADVS-10-2207536-s001.pdf]

ADVANCED SCIENCE
Open Access

Supporting Information

for *Adv. Sci.*, DOI 10.1002/advs.202207536

Nerve-on-a-Chip Derived Biomimicking Microfibers for Peripheral Nerve Regeneration

*Yunru Yu, Binghui Jin, Jinghao Chen, Chenghao Lou, Jiahui Guo, Chaoyu Yang and Yuanjin Zhao**

Supporting Information

Nerve-on-a-chip derived biomimicking microfibers for peripheral nerve regeneration

*Yunru Yu, Binghui Jin, Jinghao Chen, Chenghao Lou, Jiahui Guo, Chaoyu Yang, Yuanjin Zhao**

Experimental section:

Materials: Sodium alginate was from Aladdin. Polyvinyl alcohol (PVA, Mw ~23000) and calcium chloride (Anhydrous) were purchased from Sigma-Aldrich. A fluorescent polystyrene nanoparticle (F8811 (505/515)) was obtained from Invitrogen. GO nanosheets dispersed solution (2 mg/mL) was from XFNano. Deionized water (18.2 MΩ•cm) was achieved from Milli-Q. GelMA was self-prepared. Lithium phenyl-2,4,6-trimethyl-benzoyl phosphinate (LAP) was bought from Macklin. 184 Silicone Elastomer base and curing agent were from SYLGARD. All of the other chemical reagents were used as received, and all of the solutions were filtered before being pumped into the capillary microfluidic device.

Microfluidic spinning: The capillary microfluidic devices were assembled by coaxially aligning spindle-knotted capillary, tapered capillary, square capillary (AIT), and cylindrical capillary (WPI) on the glass slide. For the generation of hollow microfiber, one spindle capillary with an orifice of about 80 μm was inserted in a tapered capillary with an orifice of about 600 μm, both of which were regarded as injection channels. For the generation of microfiber with two or three channels, two or three spindle capillaries with an orifice of about 80 μm were inserted in the tapered capillary, respectively. The 10 wt% PVA solution, the mixture of 1.5 wt% sodium alginate and 10 wt% GelMA, and the 2 wt% calcium chloride were taken as inner, middle, and outer phases, respectively. For the generation of microfibers with GO encapsulation, different concentrations of GO nanosheet dispersions were added into the middle phase.

Rat Schwann cell cultivation: Schwann cells (RSC96) were cultured in Dulbecco's modified Eagle's medium (DMEM), added with fetal bovine serum (10%) and penicillin-streptomycin (1%), in an incubator (5% CO_2, 37 °C). The control group was the cells directly cultured on tissue culture plates, and the fiber groups were cells co-cultured with hollow microfiber and

hollow GO microfiber. The Cell Counting Kit-8 assay is used to investigate cell viability after cultivation for 24 and 72 hours. At specific time points, Schwann cells were cleansed by PBS, and 10% (v/v) CCK-8-contained fresh culture medium was added to each sample. After a 3h incubation, the absorbance was measured by a microplate reader (450 nm).

The promotion ability of nerve cell migration of microfibers was also investigated by using transwell chambers (8 μm pores, Costar). The top chambers contained 100 μL DMEM with 1×10^5 Schwann cells dispersion, and the bottom chambers were filled with 500 μL culture medium, including hollow microfibers, or GO microfibers. After being cultivated for 24 h, the upper side of the membrane was mildly cleaned, and cells adhering to the other side were treated with paraformaldehyde (4%) and stained with crystal violet (1%). The stained cells were characterized under an optical microscope.

***In vitro* degradation test:** Each sample of calcium alginate and double-network alginate/GelMA hydrogels were polymerized, dried, and weighed (W_0) before it was incubated in 500 μl PBS(pH 7.4, 0 μg/ml collagenase type 2, and pH 7.4, 5 μg/ml collagenase type 2). The samples then oscillated at 100rpm at 37 °C. At preset times, corresponding samples were finalized oscillation, cleansed by deionized water, dried, and weighed (W).

Nerve-on-a-chip: The base of silicone elastomer and its curing agent were mixed and used to replicate the designed molds of the chip. Each PDMS chip layer was polymerized in an 80 °C heater within 2 h. After cleansing the surface of the chip and treated via plasma, the microfibers were introduced to the chip. In detail, each microfiber was about 2 cm, with about 0.3 cm of two ends fixed in the middle layer channels and 1.2 cm middle part beneath the middle layer through the opening hole. The middle part of the fiber was then immobilized between the columns of the bottom layer after bonding three layers. The five channels of the bottom layer allowed fiber microfibers to be fixed and analyzed at once, and it is also possible to extend the throughput by increasing the channels of the chip. The cell suspensions (2×10^5 cells) were injected into the channel of the microfiber through the inlets of the middle layer, which are right aligned to the axis of the end fixation channels. The chip was then incubated for 1, 3, and 5 days at 37 °C. The gradient concentration of ECM was achieved by introducing 2 mg/mL and 10 mg/mL ECM dispersed culture medium into the multitrack via two inlets of the bottom layer. The Calcein-AM solution was pumped inside the microfibers on days 1, 3, and 5 to indicate the live cells. Fluorescent photographs were snapped on days 1, 3, and 5 to check the formation of nerve cell fibers.

Animal Experiment: All animal procedures received ethical approval from the Animal Experimental Committee of Wenzhou institute, University of Chinese Academy of Sciences

(WIUCAS21071206), and were conducted under animal care guidelines. 220-250 g healthy male SD rats were divided into six groups randomly: the Sham, the PNI, the silicone tube, the single-channel fiber with large channel (SCF), the dual-channel fiber (DCF), and the dual-channel GO fiber (DCGF) group. After the rats were anesthetized with pentobarbital sodium (30 mg/kg), the right sciatic nerve was exposed and a segment was removed to leave a 10 mm length of injury after the nerve stumps retracted. As for the treated groups, the removed segment was implanted with different materials. All groups were intramuscularly injected with penicillin (800,000 units) to avoid infection.

Walking track analysis: The walking track was analyzed at 2, 4, and 8 weeks after the surgery when they were guided in a narrow and straight space with their hind paws treated with non-toxic inks. Several parameters of footprints were measured to analyze the SFI, including the footprint length on experimental and normal sides (EPL, NPL), distance from toe 1 to toe 5 on experimental and normal sides (ETS, NTS), and that between toes 2 and 4 on experimental and normal sides (EIT, NIT). The SFI was measured based on the formula shown in Figure S12. The value approaching 0 indicated a good recovery, but reaching -100 suggested a total injury.

Electrophysiology: Electrophysiological analyses were implemented to study the function recovery in the 8th week. After the rats were anesthetized, the sciatic nerves were exposed their proximal site, where an electrode was attached to emit electrical signals. In this procedure, another electrode was attached to the gastrocnemius belly on the same side to form the electromyograph. The compound muscle action potentials (CMAPs) were recorded, and the amplitude, latencies, and conduction velocities of different groups were analyzed.

Histological analysis of the gastrocnemius muscle and regenerated nerve: The animals were sacrificed 8 weeks after the surgery, and the rebuilt nerves at the right side as well as gastrocnemius muscles at both sides were collected. After 24 h fixation in 4% paraformaldehyde, tissues were embedded in paraffin or OCT. The muscle cross-sectional slices were stained by H&E together with Masson trichrome to examine the CSA and pathologic changes. The longitudinal nerve sections were then stained according to the protocol: chicken anti-NF200 (1:1000), and rabbit anti-S100β (1:300). The transverse nerve sections were stained by chicken anti-NF200 (1:1000), and rabbit anti-MBP (1:300). After being incubated with fluorescent secondary antibodies (1:1000), slices were treated with DAPI Fluoromount-GTM.

Characterization: Microfibers and the nerve-on-a-chip was observed under the stereomicroscope (Olympus, SZX16). The online observation of microfluidic spinning was achieved under a fast camera (Pike, F032B). Fluorescent microfibers were also characterized under a confocal laser microscope (Nikon, A1). The SEM images were taken from a field

emission scanning electron microscope (HITACHI, SU8010). The conductivity of the microfiber was studied via a digital multimeter (Keithley, DMM6500) by using a traditional two-probe manner. All images of histological assessments were captured on a Nikon Eclipse 80i, or a confocal laser microscope (Nikon, Ti-E&A1 plus).

Statistical analysis: All data in the experiment were expressed as mean ± standard deviations. Statistical analysis was evaluated using Origin. Statistical evaluation was analyzed using one-way ANOVA and the differences were considered statistically significant at the value of $*p < 0.05$, $**p < 0.01$, or $***p < 0.001$.

Supporting Figures:

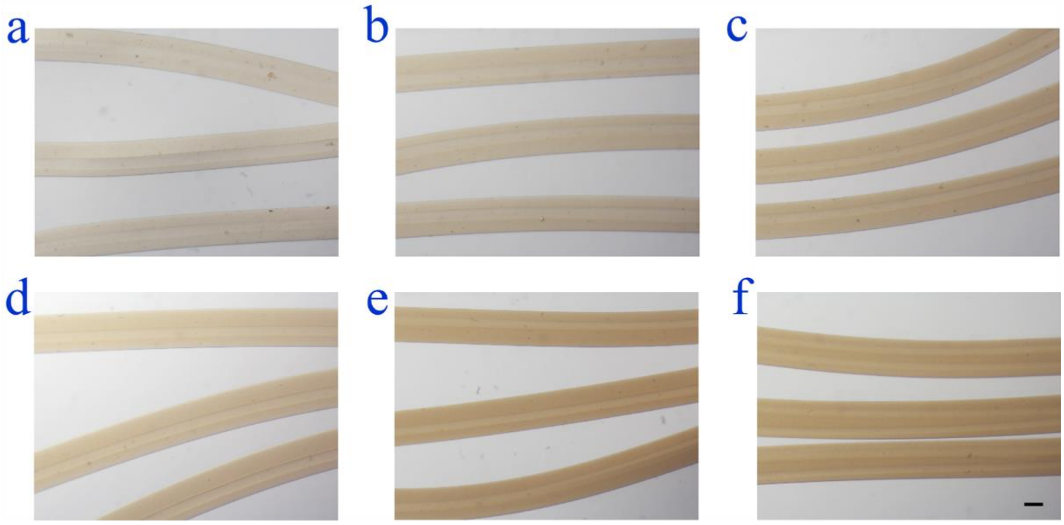

Figure S1. Optical microscopic images of microfibers with different concentrations of GO and morphologies. (a) Hollow microfiber with 2 mg/mL GO encapsulation. (b) Hollow microfiber with 4 mg/mL GO encapsulation. (c) Hollow microfiber with 6 mg/mL GO encapsulation. (d) Hollow microfiber with 8 mg/mL GO encapsulation. (e) Hollow microfiber with 10 mg/mL GO encapsulation. (f) Dual-channel microfiber with 10 mg/mL GO encapsulation. The scale bar is 200 μm.

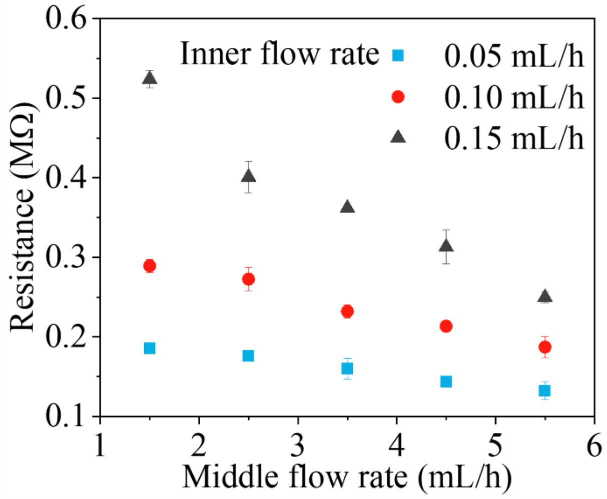

Figure S2. The relationship between the resistance of microfiber and the generation flow rates.

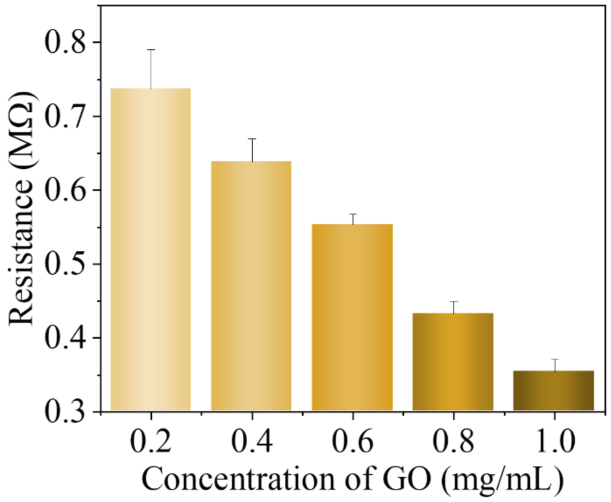

Figure S3. The relationship between the resistance of microfiber and the concentration of GO encapsulated in the microfiber.

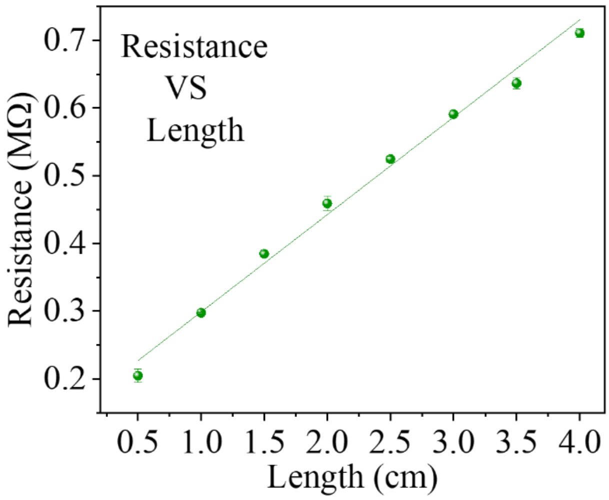

Figure S4. The relationship between the resistance of microfiber and the fiber length.

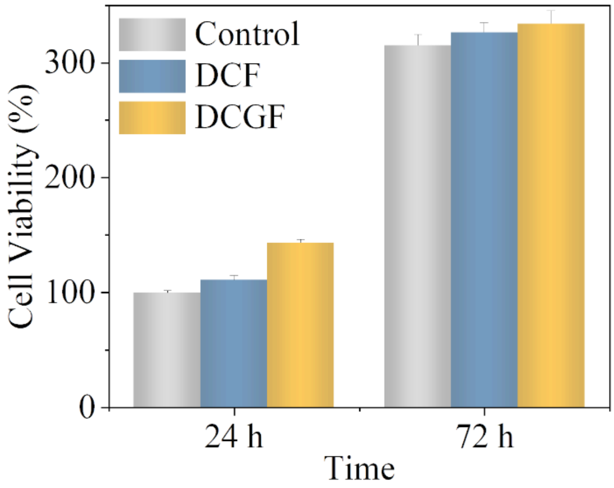

Figure S5. The cell viability after 24h and 72h incubation with different kinds of microfibers.

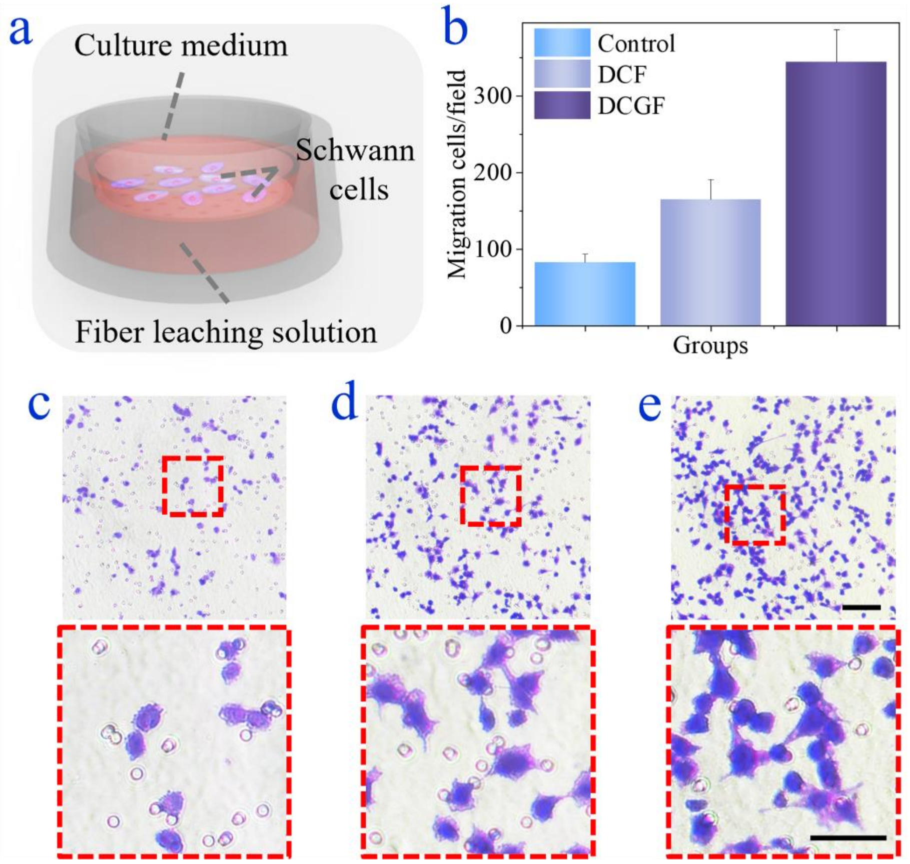

Figure S6. Schwann cell migration assay. (a) Scheme of experimental design for Schwann cells migration assay. (b) Statistical analysis of the migration of Schwann cells per field for each group. (c-e) Microscopic images of migrated Schwann cells in (c) control, (d) DCF, and (e) DCGF group. Scale bars are 100 μm, and 50 μm, respectively.

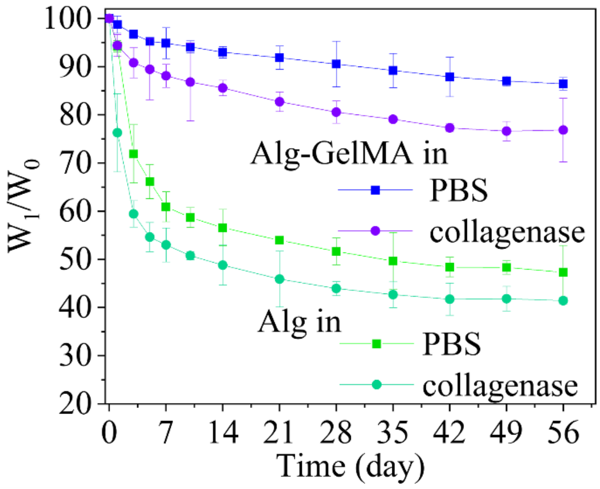

Figure S7. Degradability characterization.

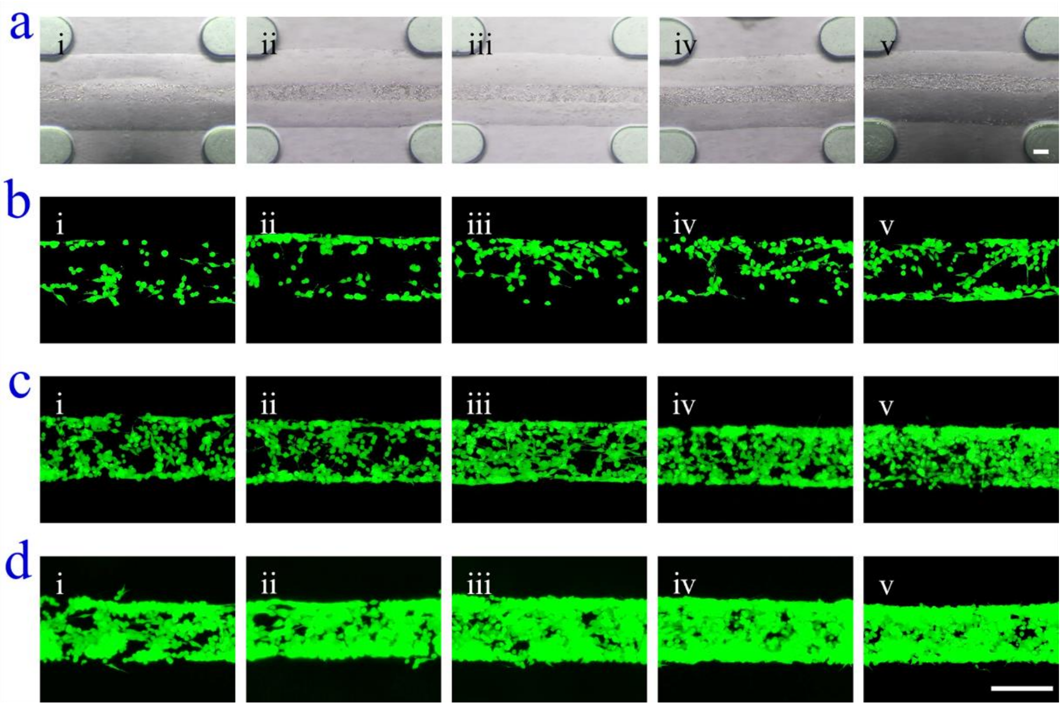

Figure S8. Cell proliferation of microfibers in a gradient ECM cultivation medium. (a) Optical microscopic images of cells cultivated in a gradient concentration of ECM. The concentration increased from (i) to (v). (b-d) Representative fluorescent images of formed cell bundles on (b) Day 1, (c) Day 3, and (d) Day 5. The scale bars are 200 μm.

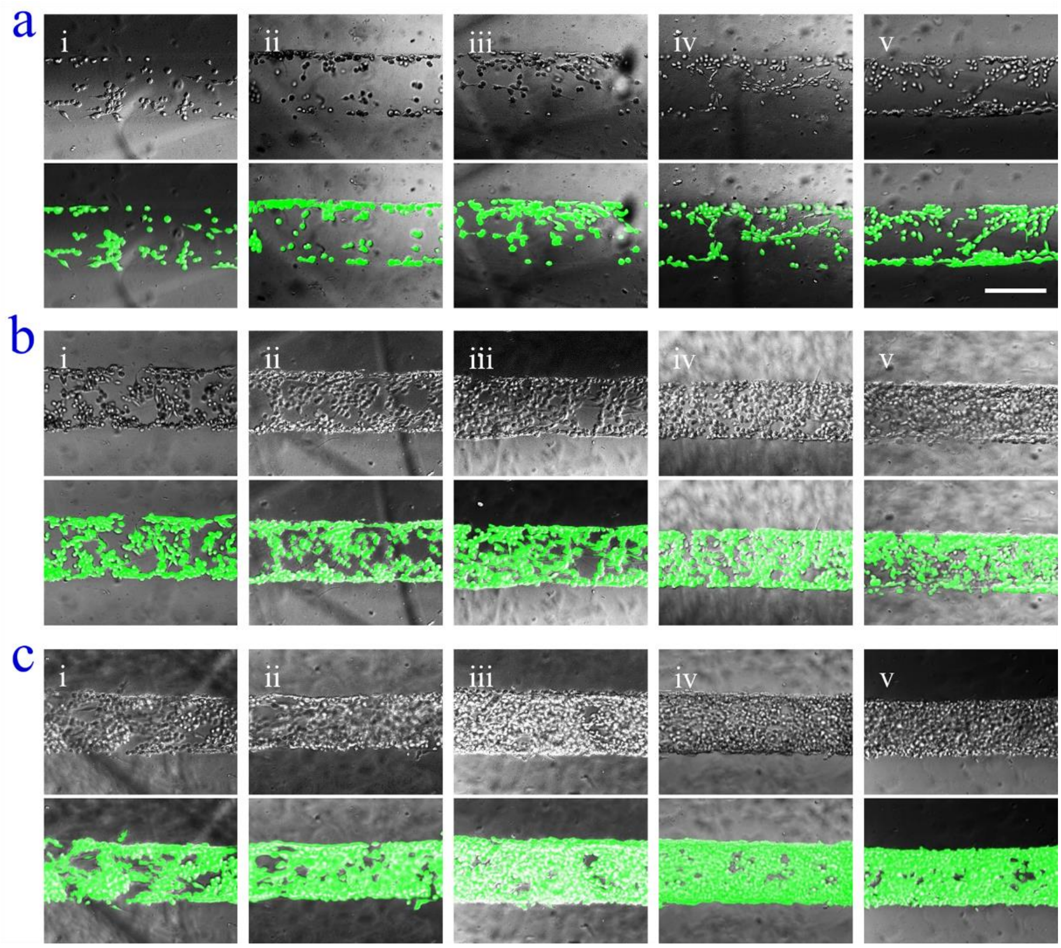

Figure S9. Cell proliferation of microfibers in a gradient ECM cultivation medium. (a-c) Confocal laser scanning images in bright-field and merged channels of formed cell bundles corresponding to Figure S8 b-d. The scale bar is 200 μm.

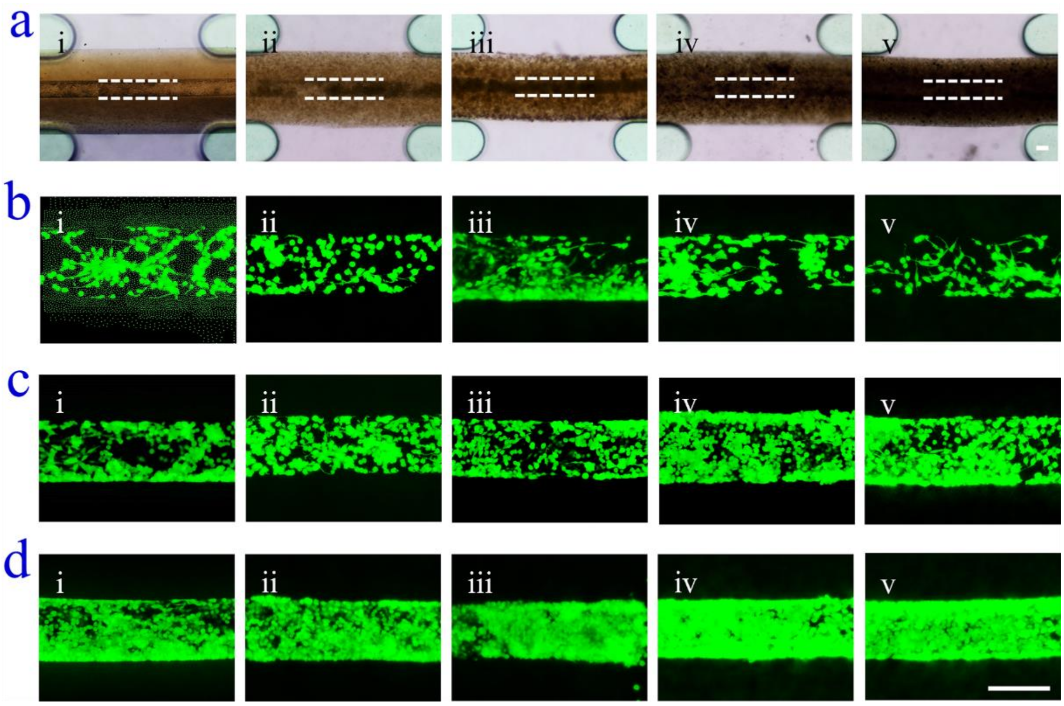

Figure S10. Cell proliferation of microfibers in microfibers with an increase of GO encapsulation. (a) Optical microscopic images of cells cultivated in microfibers with different concentrations of GO. The concentration increased from (i) to (v). (b-d) Representative fluorescent images of formed cell bundles on (b) Day 1, (c) Day 3, and (d) Day 5. The scale bars are 200 μm.

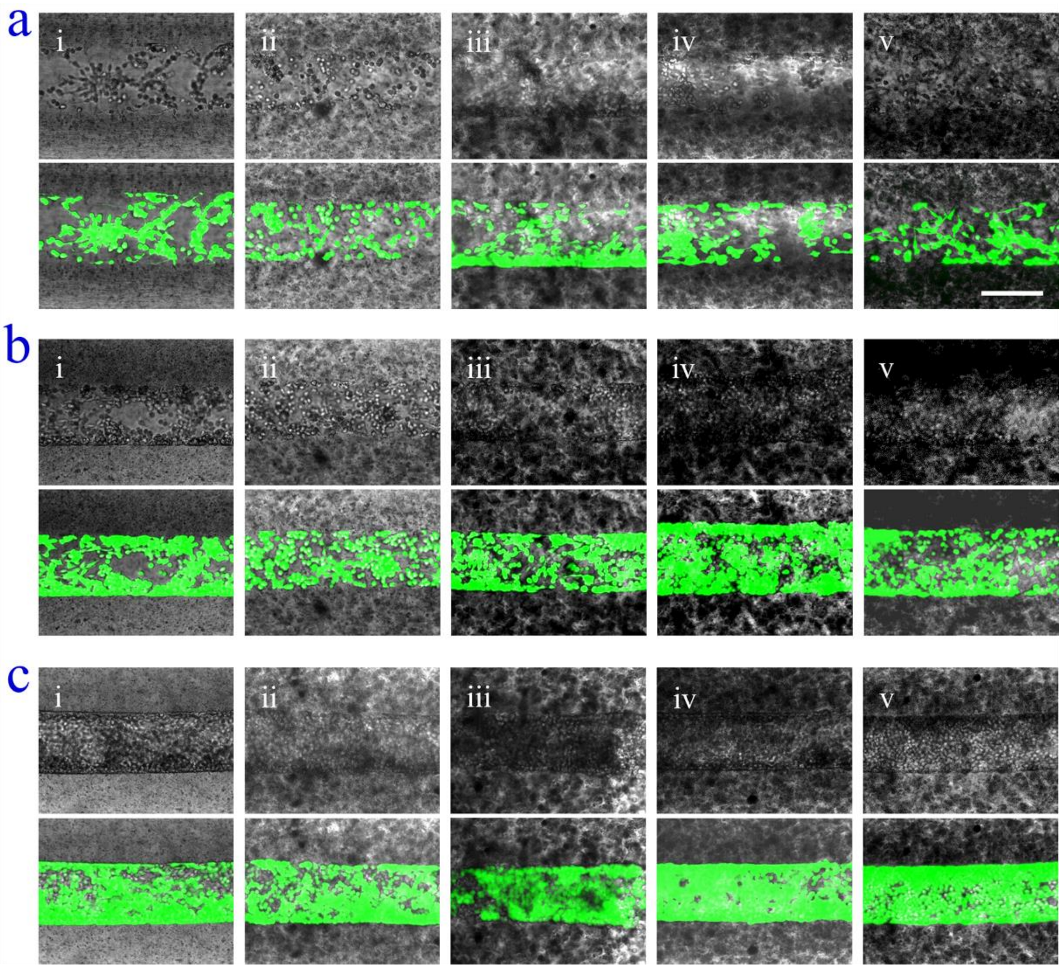

Figure S11. Cell proliferation in microfibers with an increased concentration of GO encapsulation. (a-c) Confocal laser scanning images in bright-field and merged channels of formed cell bundles corresponding to Figure S10 b-d. The scale bar is 200 μm.

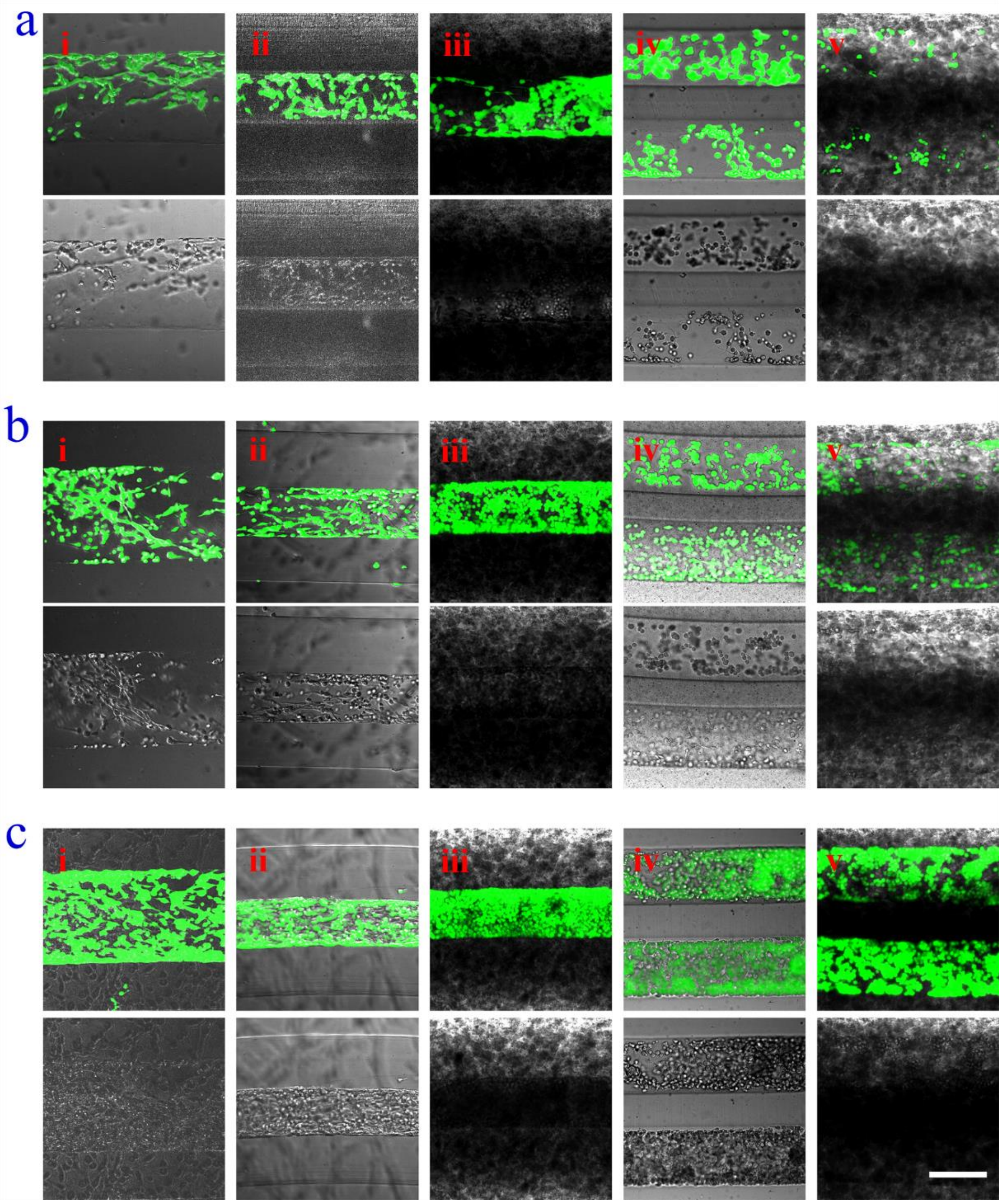

Figure S12. Cell proliferation in microfibers with different morphologies. (a-c) Confocal laser scanning images in bright-field and merged channels of formed cell bundles corresponding to Figure 4 c-e. The scale bar is 200 μm.

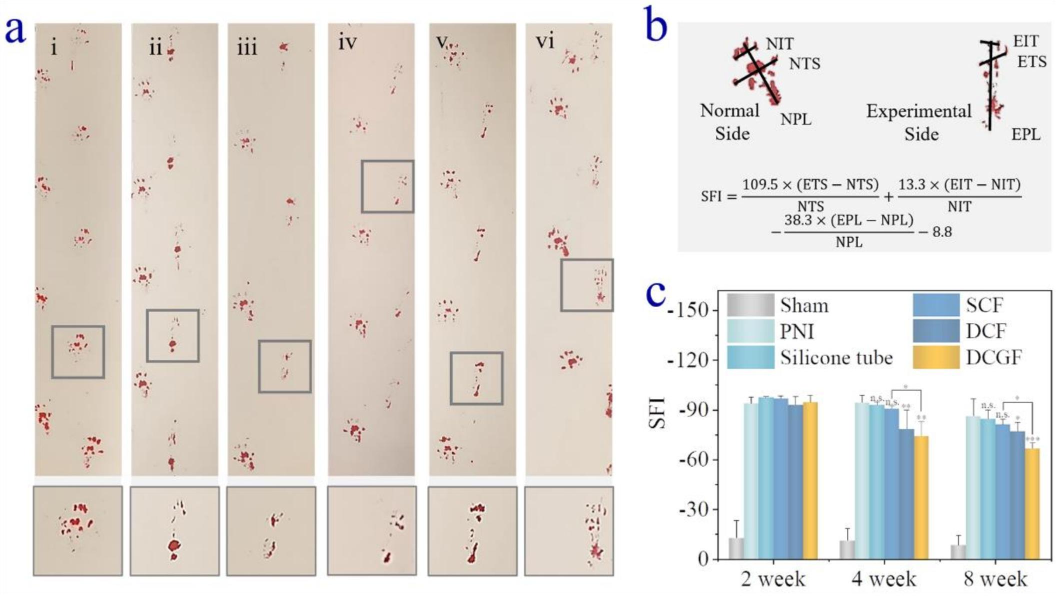

Figure S13. SFI analysis. (a) Representative images of rat footprints in (i) Sham, (ii) PNI, (iii) Silicone tube, (iv) SCF, (v) DCF, and (vi) DCGF groups at 8 weeks post-surgery. (b) Scheme of parameters in SFI study and the formula of SFI calculation. (c) SFI analysis at 2, 4, and 8 weeks after surgery. (n⩾3, $*p < 0.05$, $**p < 0.01$, $***p < 0.001$, n.s.: not significant, versus PNI).

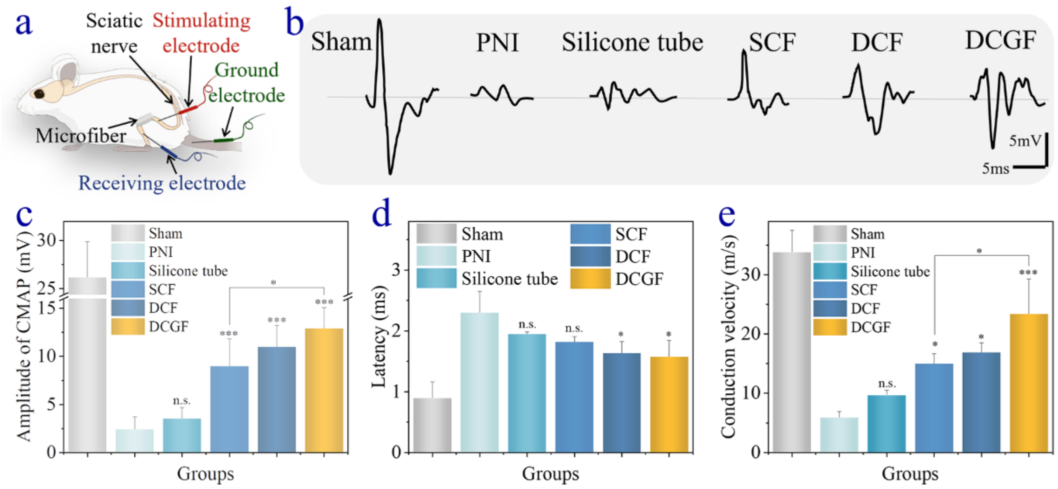

Figure S14. Electrophysiology study. (a) Schematic illustration of electrophysiology analysis manner. (b) Represent CMAP records in each group. (c-e) The statistical analysis of (c) the amplitude of CMAP, (d) latency, and (e) conduction velocity in each group. (n⩾3, *$p < 0.05$, **$p < 0.01$, ***$p < 0.001$, n.s.: not significant, versus PNI).

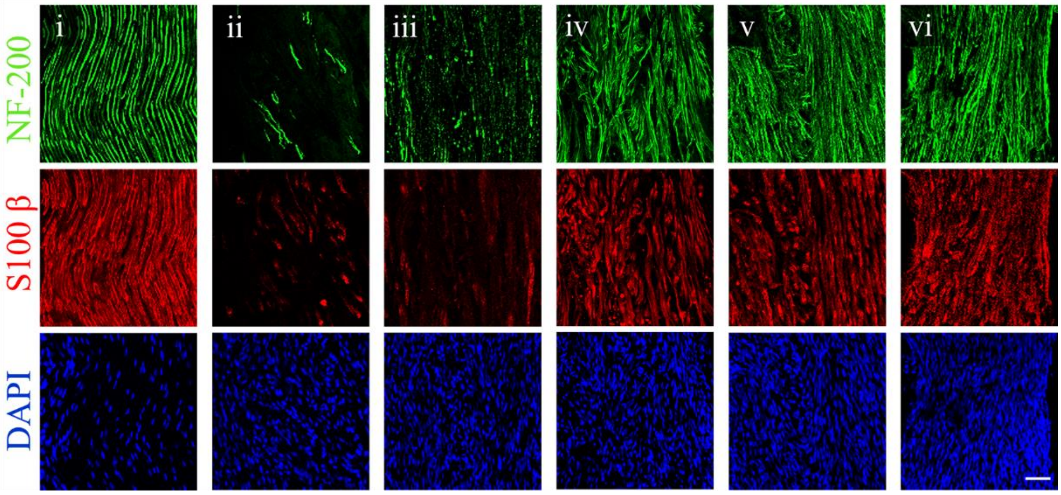

Figure S15. Representative images of NF-200, S100β, and DAPI of longitudinal sections of regenerated nerves in (i) sham, (ii) PNI, (iii) Silicone tube, (iv) SCF, (v) DCF, and (vi) DCGF 8 weeks post-surgery. The scale bar indicates 50 μm.

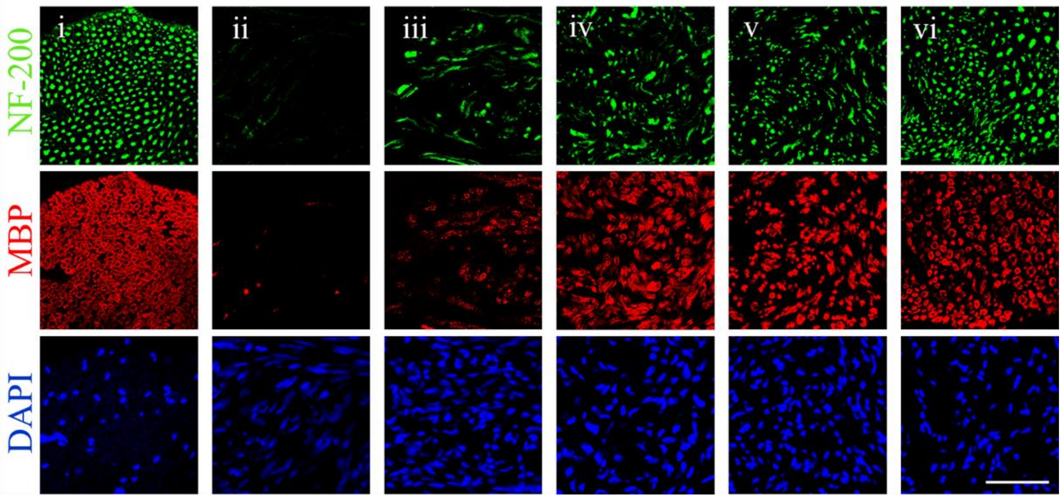

Figure S16. Representative immunofluorescence images of NF-200, MBP, and DAPI of cross-sectional sections of regenerated nerves in (i) sham, (ii) PNI, (iii) Silicone tube, (iv) SCF, (v) DCF, and (vi) DCGF 8 weeks post-surgery. The scale bar is 50 μm.

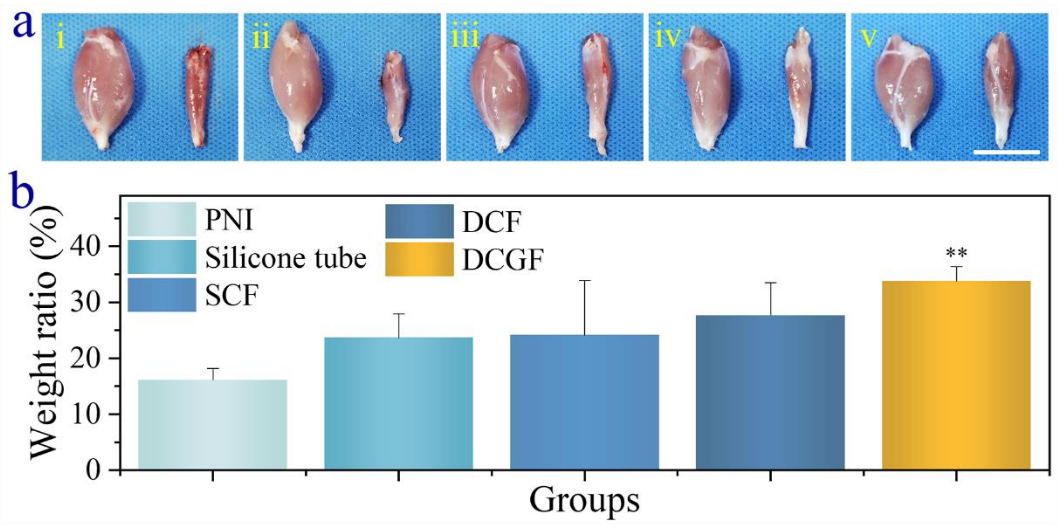

Figure S17. The weight ratio of the gastrocnemius muscle in each treatment group. (a) Digital photographs of bilateral gastrocnemius muscle in (i) PNI, (ii) Silicone tube, (iii) SCF, (iv)DCF, and (v) DCGF group. (n⩾3, ***p* < 0.01, versus PNI).
